# Exposure to Endosulfan can result in male infertility due to testicular atrophy and reduced sperm count

**DOI:** 10.1038/cddiscovery.2015.20

**Published:** 2015-11-09

**Authors:** R Sebastian, SC Raghavan

**Affiliations:** 1 Department of Biochemistry, Indian Institute of Science, Bangalore, India

## Abstract

Endosulfan (ES) is a widely used organochlorine pesticide and is speculated to be detrimental to human health. However, very little is known about mechanism of its genotoxicity. Using mouse model system, we show that exposure to ES affected physiology and cellular architecture of organs and tissues. Among all organs, damage to testes was extensive and it resulted in death of different testicular-cell populations. We find that the damage in testes resulted in qualitative and quantitative defects during spermatogenesis in a time-dependent manner, increasing epididymal reactive oxygen species levels, affecting sperm chromatin integrity. This further culminated in reduced number of epididymal sperms and actively motile sperms. Finally, we show that ES exposure affected fertility in male but not in female mice. Therefore, we demonstrate that ES exerts pathophysiological changes in mice, induces testicular atrophy, affects spermatogenesis, reduces quantity and vigour of epididymal sperm and leads to infertility in males.

## Introduction

Pesticides encompass a wide range of chemicals used to kill insects, weeds or fungi for better yields in organised farming. Many a time, these benefits come at the cost of adverse effects on the environment and non-target organisms including humans.^1^ Among various classes of pesticides known to cause side effects, organochlorine pesticides (OCPs) lead the list, possessing high transport potential, and a variety of toxic and untoward health effects.^2,3^ Endosulfan (ES; 6,7,8,9,10,10-hexachloro-1,5,5a,6,9,9a-hexahydro- 6,9-methano-2,4,3-benzodioxathiepin-3-oxide), a cyclodiene OCP consisting of *α* and *β* isomers (3 : 1), is of special interest as a persistent environmental pollutant^4–6^ with speculated adverse effects on humans.^7^

World health organization (WHO) has classified ES as a Class II pesticide (moderately toxic) and it is listed under the Stockholm convention as a persistent organic pollutant, given its environmental impact (http://chm.pops.int/default.aspx). Due to its extensive usage spanning over 50 years, it is one of the most commonly detected pesticide in surface waters in USA without a declining trend and a much abundant OCP in the air.^8,9^ Although phased out in some countries, ES is still widely used in several Asian countries including India and China, subjecting ~40% of world population to its direct effects^10–13^ and is of worldwide concern due to increasing global trade of farm produce. Despite its association with many putative abnormalities and birth defects in humans in areas of extensive use, there are no molecular studies to investigate its mechanism of action in causing cellular damage, genomic instability and ill-health.

There are limited studies thus far that investigate mechanism of action of ES in mammals.^7,14–16^ Previous investigations have failed to explain molecular basis of deformations and abnormalities associated with ES exposure in humans.^17,18^ Besides, the precise mechanisms by which ES exerts its effects remains unclear and studies on its role in inducing cellular damage are limited.

Here, using physiologically relevant concentrations of ES, we report that ES exerts distinct and specific pathophysiological changes in mice, affecting liver function and perturbing blood cell numbers. It exerts tissue damages in lungs and liver and causes acute atrophy in seminiferous tubules of testes. Further, the damage in testicular cells leads to cell death, affecting spermatogenesis in male mice, resulting in severe reduction in sperm count and motility leading to infertility. Thus, our study demonstrates the mechanism of ES-mediated testicular toxicity and male infertility.

## Results

### ES affects normal physiology in mice

**Figure 1:**
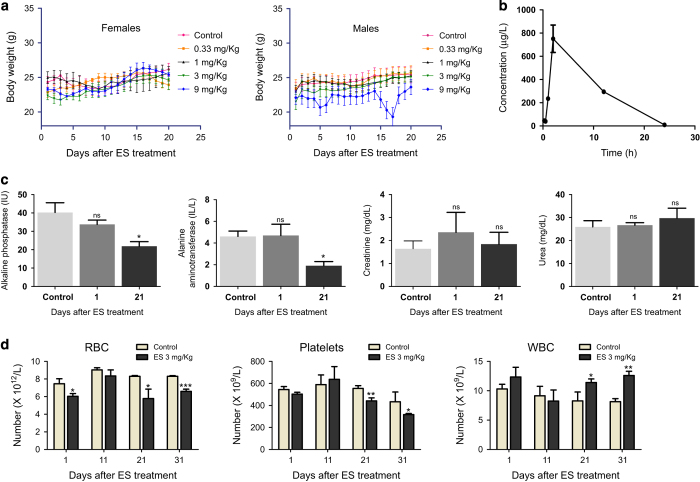
Evaluation of physiological effects of Endosulfan in mice. (**a**) Body weight distribution of the ES-treated animals (*n*=5 per group) following exposure to different doses of Endosulfan (0, 0.33, 1, 3 and 9 mg/kg body weight). Mean body weight of the group is plotted against days after the offset of treatment. (**b**) Graph derived from the area of the peak in HPLC profile, showing the bioavailability of ES in mice after oral ingestion of ES (3 mg/kg) against time. (**c**) Bar graphs indicating enzymatic activities reflective of liver and kidney function at 1 and 21 days after ES treatment (3 mg/kg, *n*=5). Alkaline phosphatase and Alanine aminotransferase are markers of liver function; urea and creatinine are of kidney function. IU corresponds to international unit. *n*=5 in all groups. (**d**) Bar graphs showing RBC, WBC and platelet counts at different time points (1, 11, 21, 31 days) after ES treatment (3 mg/kg) completion. NS, not significant.

To evaluate the pathophysiological changes induced by ES exposure, we assessed various responses in mice following ES treatment. Body weight fluctuation is an important parameter in understanding the physiological effects of a compound. Body weight analyses of male and female mice following ES treatment (0, 0.33, 1, 3, 9 mg/kg) for a period of 20 days showed remarkable fluctuations in weight in a concentration-dependent manner in case of male mice, while there was no significant change in females (Figure 1a). Thus, our results suggest that male mice are more sensitive to ES as compared with females and therefore male mice were chosen for further studies.

Choosing the appropriate concentration of ES for analysis of physio-molecular changes is the key to correlate the observations made in model systems with humans. To ascertain serum concentration of ES in mice, we performed HPLC analysis of serum samples collected at different time points (0.25, 0.5, 1, 2, 6, 12 and 24 h) after oral ingestion (3 mg/kg) (Supplementary Figure S1A). ES-spiked serum served as standard. Results showed that ES peaked in serum at 2 h with a concentration of 700 *μ*g/l and then showed a gradual decrease and by 24 h, dropped to 23 *μ*g/l (Figure 1b, Supplementary Figure S1A). The peak concentration was well below the lethal concentration observed in case of ES poisoning in humans (Supplementary Figure S1B). Analysis of data from previous studies on ES concentrations detected in serum collected from human subjects living in areas of exposure showed that the concentration levels were comparable to what we have used in the current study (Supplementary Figure S1C). Hence, the ES concentration we have chosen for our studies in mice (3 mg/kg) is physiologically relevant and comparable to the levels present in humans under active involuntary exposure.

Liver function tests were performed to evaluate further biochemical changes in the organs. Results showed decreased levels of alkaline phosphatase (ALP), a hydrolase enzyme of liver, on days 1 and 21 after ES treatment indicating acute hepatotoxicity (Figure 1c). Besides, alanine amino transferase (ALT), an enzyme involved in production of pyruvate and L-glutamate by transamination reaction, was reduced at day 21 following treatment. Although no difference was noticed immediately after treatment, a reduced ALT at a later time post treatment is indicative of hepatocellular injury, which was evident in histological analysis of liver of ES-treated mice. However, analysis of kidney function (urea and creatinine levels) suggests that it was largely unaffected following ES treatment (Figure 1c).

We have investigated the effects on RBC, WBC and platelets following exposure to ES in mice (3 mg/kg). The quantitative analysis at 1, 11, 21 and 31 days post-ES treatment showed decreased levels of RBC and platelets, which was also reported in humans following accidental ES ingestion (Figure 1d).^19,20^ Interestingly ES treatment led to increased levels of WBC, possibly due to an inflammatory reaction.

To test whether immune effector cells are compromised in ES-treated mice, we analysed CD3^+^ (T cell marker) cell population in thymus and CD19^+^ (B cell marker) cell population in bone marrow, following treatment (3 mg/kg, 1st day). Although the mean percentage did not vary for thymic and bone marrow populations, there was a marked difference in the CD19^+^ population, following treatment with ES as compared with controls, suggesting toxicity in a sub group of animals (Supplementary Figure S1D). However, such a difference was not seen for CD3^+^ cells (Supplementary Figure S1D).

### ES induces tissue damage in testes and other organs

**Figure 2:**
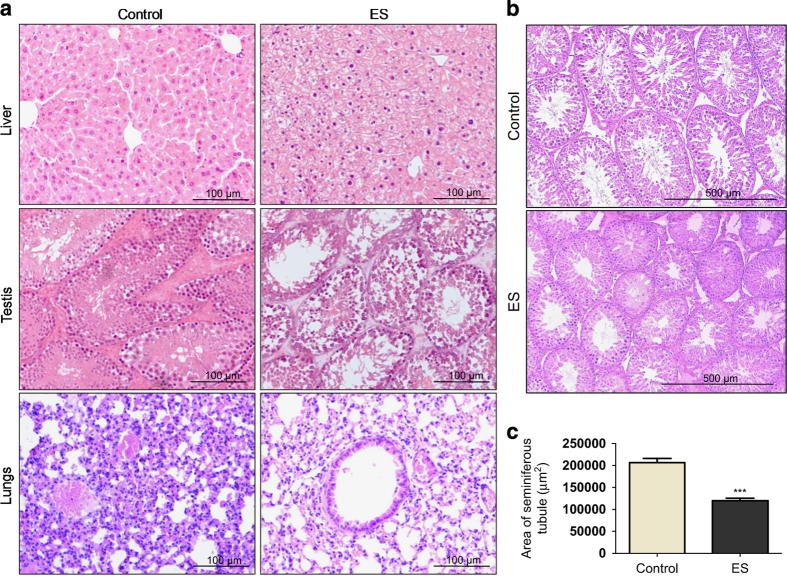
Histopathological examination of organs from Endosulfan exposed mice and rat. (**a**) Histopathology of liver, testes and lungs of mice following ES treatment. Control in all panels indicates respective tissues from mice with no treatment and ES represents tissues from ES-treated mice (20×) (3 mg/kg, 1st day). (**b**) Histopathology of rat testes after ES treatment (10×) (3 mg/kg, 1st day). (**c**) Bar graph showing the relative difference in the diameter of seminiferous tubules of testes from control group and Endosulfan-treated rats (3 mg/kg). *n*=20 tubules in each group.

To understand the direct effect of ES on histopathology of vital organs, histology of liver, testes, brain, kidney, intestine and lungs was performed following ES treatment on mice (3 mg/kg, 1st day). Results showed that liver, testes and lungs were affected maximally on ES treatment (Figure 2a) while brain, kidney and intestine did not exhibit any significant difference when compared with controls (Supplementary Figure S2A). Analyses of liver sections revealed necrotic liver hepatitis and hepatic congestion following ES treatment indicating acute hepatic toxicity (Figure 2a). Testes showed high degree of atrophy and tubular necrosis, in which many seminiferous tubules were affected with fully or partially depleted spermatogonial mother cells and spermatids. A visible difference was also seen in sperm population in affected seminiferous tubules (Figure 2a). Interstitial cells were also extensively defoliated (Figure 2a). We also found significant differences in ES-treated lungs as compared with untreated controls (Figure 2a). Luxol blue staining of the brain sections from ES-treated and control mice did not show any difference, suggesting intact brain anatomy (Supplementary Figure S2B).

Since we observed the significant difference in mice testes physiology on ES treatment, we wondered whether this observation holds true in case of other model systems. Histological analysis of the wistar rat testes at identical ES concentration and time period showed significant reduction in the area of seminiferous tubules with fully or partially depleted spermatogonial mother cells and spermatids, indicating testicular damage (Figures 2b and c).

### Exposure to ES leads to cell death in different testicular cells

As ES-induced hepatic toxicity is associated with the altered metabolic activities following pesticide exposure, lungs and testes were chosen for further investigation to understand the mechanism of ES action.

**Figure 3:**
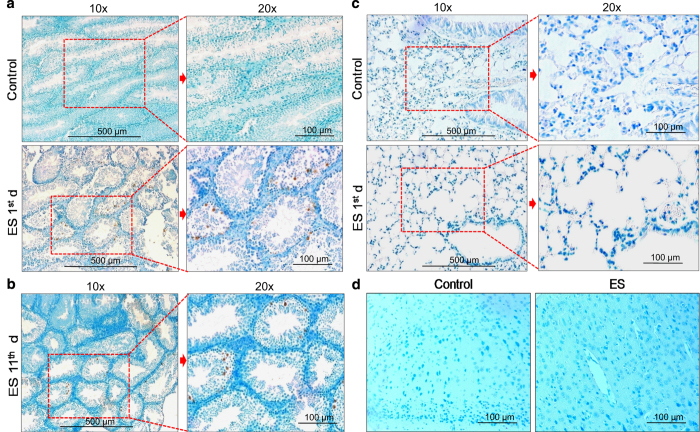
Endosulfan-induced cell death is specific to testes. (**a**) TUNEL assay showing DNA fragmentation in testicular cells following ES treatment (3 mg/kg) in male mice. Green colour indicates methyl green counter-stained nuclei and the brown spots are broken DNA stained with diaminobenzidine (DAB). Control sections are devoid of TUNEL-positive cells. (**b**) TUNEL assay performed on sections obtained at 11th day post-ES treatment showing TUNEL-positive cells. indicating persisting cell death. (**c**) TUNEL assay showing no stained cells in control and ES-treated mice lungs (1st day). (**d**) TUNEL assay in brain sections showing absence of positively stained cells (×20).

To assess whether ES induces cell death in testes, TUNEL assay was performed on testicular section of mice treated with ES (3 mg/kg; 1, 11 day). Results showed TUNEL-positive cells among spermatogonial mother cells, spermatocytes and spermatids 24 h post-ES treatment (Figure 3a). Interestingly, despite pathophysiological changes, DNA damage response and changes in the levels of apoptotic proteins (RS and SCR, unpublished observations), we could not observe TUNEL-positive cells in lungs on ES treatment (Figure 3c). This could be due to the limitation of scoring apoptosis in lungs as apoptotic cells would be cleared much efficiently and swiftly in lungs than in other organs.^21^ TUNEL-positive cells were repeatedly observed in testes, when experiment was repeated 11 days after ES treatment, suggesting persistent DNA damage (Figure 3b). Brain sections did not show any sign of apoptosis following ES treatment, justifying the observed intact architecture in histological studies (Figure 3d).

### ES-induced testicular-cell death perturbs spermatogenesis

**Figure 4:**
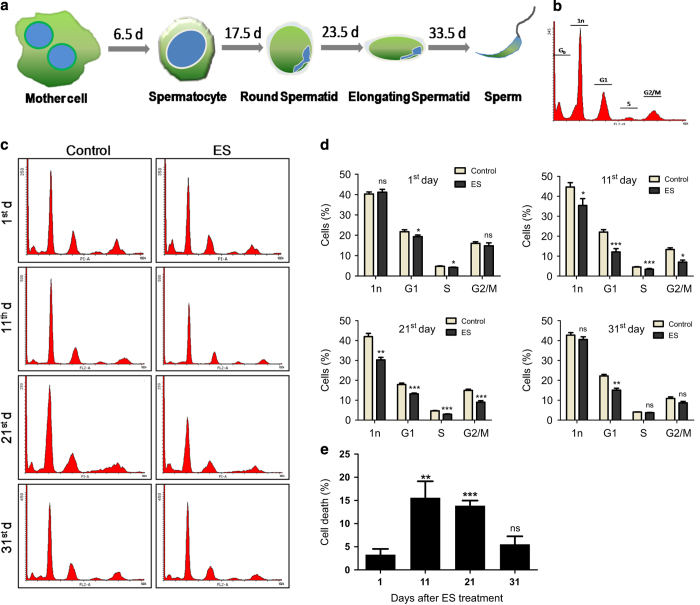
Evaluation of changes during spermatogenesis following ES exposure. (**a**) Schematic showing different stages of spermatogenesis in mice. (**b**) Representative testicular FACS profile of an untreated mouse with cell cycle phases marked. 1n indicates round, elongating and elongated spermatids, which constitutes the majority of cells in testis. G1 peak consists of somatic cells in testis, secondary spermatocytes and spermatogonial mother cells at their G1 phase. S and G2/M phases are constituted solely by the mother cells at their different divisional phases, since somatic cells in testis do not divide. (**c**) Representative FACS analysis of PI-stained testicular cells from ES-treated mice (3 mg/kg) at different time points after treatment completion showing G_0_, 1n, G1, S and G2/M populations. (**d**) Graphs indicate the time-dependent changes in testicular-cell populations on ES treatment obtained using FACS analysis (3 mg/kg, *n*=5 in each group). Separate controls were acquired along with samples belonging to different time points. 1n and G1 represent spermatids and diploid cells, respectively. S and G2/M phases indicate spermatogonial mother cells undergoing division. (**e**) ES-induced, time-dependent testicular-cell death, relative to the control obtained from testicular FACS analysis (*n*=5 per group).

In mice, spermatogenesis follows a progressive pattern of cell division, from spermatogonial mother cells, situated at basal epithelial layer of seminiferous tubules to elongated spermatocytes, which are stored in the lumen and later transported to epididymis (Figure 4a).^22^ Since spermatogenesis is a time-dependent event, perturbation in any of its cell population in the seminiferous tubule would result in a whiplash response in the succeeding cell populations. Therefore, we were interested in investigating different testicular-cell populations in a time-dependent manner, following ES treatment (3 mg/kg; 1, 11, 21, 31 day). FACS analysis was performed on testicular cells using propidium iodide (PI) staining (Figure 4b). At each time point, controls were assayed along with experimental group and percentage of cells belonging to each stage of spermatogenesis was calculated. Results showed remarkable reduction in cell populations at G1 and S phase after 24 h of ES treatment (Figures 4c and d, Supplementary Figures S3A and B). On 11th day post-ES treatment, reduction in G1 and S phase population became more prominent leading to subsequent reduction in G2/M (4n) and 1n (spermatids) cells. A significant reduction in spermatids was observed on 21st day indicating the whiplash effect of mother cell depletion observed on day 11. Interestingly, on day 31, most of the cell populations were restored to the levels equivalent to controls, except the diploid cell population. These results indicate that ES treatment significantly perturbed a complete cycle of spermatogenesis, causing testicular atrophy and depletion of cell populations.

FACS analysis of sub G1 population following ES treatment showed very limited cell death after 1 day of ES treatment, while it was maximal at day 11, and reduced thereafter, supporting the above described depletion in cell number at various phases of spermatogenesis (Figure 4e). The observed highest cell death at day 11 was also reflected in the dip observed in G1, G2/M and 1n populations (Figure 4d). G_0_ population on day 31 was comparable to that of day 1, confirming the reversal of the effect after one complete cycle of spermatogenesis. Taken together, FACS analysis of murine testes demonstrates the ES-mediated testicular-cell depletion in a spermatogenesis-dependent manner.

### ES affects the integrity of sperm in male mice

**Figure 5:**
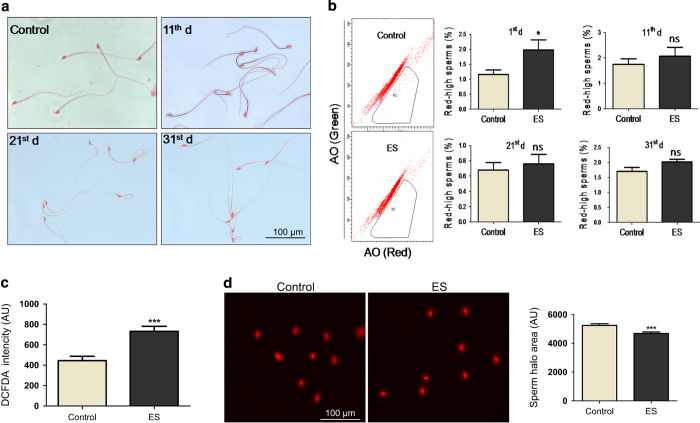
Evaluation of effect of ES on epididymal sperm integrity. (**a**) Microscopic analysis of sperm morphology on ES exposure (3 mg/kg) at different time points (0, 10, 20 and 30 days) post-treatment completion. Samples are stained with Eosin Y. (**b**) Representative FACS analysis of sperm chromatin structure assay. P2 represents red-high sperm indicating loss of chromatin integrity in epididymal sperm cells. Bar graphs show cumulative red-high sperm obtained from ES-treated mice (3 mg/kg) at various time points (10, 20 and 30 days; *n*=5 per group). (**c**) Bar graphs indicating the epididymal sperm ROS levels following exposure to ES (3 mg/kg) measured using DCFDA staining followed by FACS (*n*=9 and 11 for control and ES, respectively). (**d**) Propidium Iodide-stained sperm heads after sperm dispersion assay (halosperm assay) performed on the epididymal sperm sample that exhibited highest ROS (detected in FACS) following treatment with ES (3 mg/kg). Bar graphs indicate the quantitative representation of the sperm halo area in control and treated groups (*n*=210 and 179 in control and treated, respectively).

Based on above results, we wondered whether ES exerts any effect on sperm morphology and integrity. To address this, epididymal sperm was collected from mice following treatment with ES (3 mg/kg, 11, 21, 31 day), subjected to Eosin Y staining and analysed. Compared with untreated control, we could not observe any difference in the morphology of hook, head and tail of the sperms (Figure 5a), revealing that ES may not have a direct impact on epididymal sperm morphology.

To check whether ES treatment affects chromatin integrity of epididymal sperms, sperm chromatin structure assay (SCSA) was performed. Results showed that the percentage of red-high sperms increased significantly on 1st day post treatment with ES (3 mg/kg). However, there was no significant difference between treated and untreated groups observed on day 11, 21 and 31 (Figure 5b), suggesting that compromised DNA integrity in epididymal sperm could be directly related to the bioavailability of ES within the cells.

Reactive oxygen species (ROS) are potential threats to the genome and are responsible for generating DNA single- and double-strand breaks.^23^ One of the important factors responsible for compromised sperm DNA integrity is elevated levels of ROS. Since we observed reduced sperm chromatin integrity within epididymal sperm, we wondered whether this could be due to ROS. We performed DCFDA-mediated FACS analysis of epididymal sperm collected on 1st day post-ES treatment (3 mg/kg), and results showed that the ROS level in treated group was significantly higher than that of control (Figure 5c). This suggests that ROS could be responsible for the transient increase in red-high sperm contributing to loss of DNA integrity. To further investigate this, we performed sperm chromatin dispersion assay using epididymal sperm, which showed highest ROS in the DCFDA staining. Results showed a reduction in halo formation in ES-treated sperm, indicative of broken DNA and therefore less puffing in the lysis solution, suggesting reduced levels of DNA integrity (Figure 5d). Taken together, these results indicate that ES exerts a direct transient effect on epididymal sperm immediately after exposure, which is mediated through elevated ROS levels, contributing to genomic instability within testes.

### ES reduces epididymal sperm count and quality

**Figure 6:**
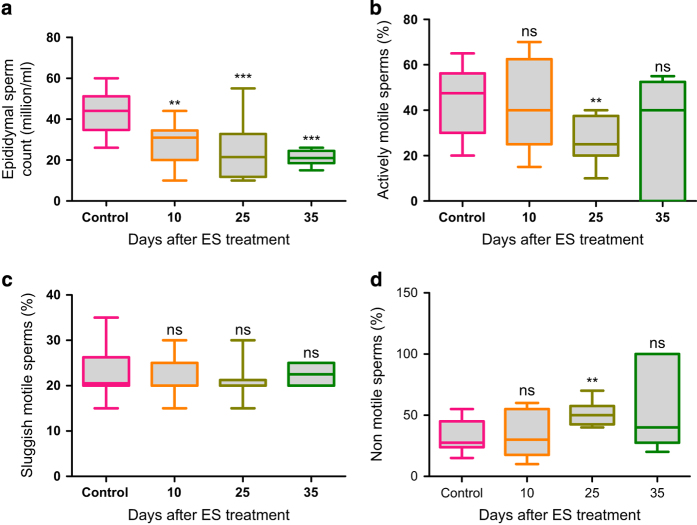
Quantitative analyses of epididymal sperms on Endosulfan exposure. (**a**–**d**) Analyses of epididymal sperm collected from ES-treated mice (3 mg/kg) at various time points (11, 26 and 36 days) after treatment completion (*n*=9 and 10 for control and treated, respectively) showing total sperm count (**a**), actively motile sperms (**b**), sluggish motile sperms (**c**) and non-motile sperms (**d**). NS, not significant.

To check the effects of spermatogenesis-dependent testicular-cell depletion on epididymal sperm following treatment with ES (3 mg/kg, 11, 26 and 36 day), clinical analysis of epididymal sperm was carried out post-ES treatment. The time points were selected based on the completion of the mating windows used in the experiment explained below. We found a reduction in epididymal sperm count on day 11 post treatment. The difference was prominent and significant when the sperm count was taken on day 26 and 36 post-ES treatment, suggesting that effect of ES on spermatogenesis contributed to a dramatically reduced sperm count (Figure 6a). Although there was no increase in sluggish motile sperms, significant reduction in actively motile sperms and increase in non-motile sperms was observed particularly on day 26, a probable consequence of testicular-cell damage and death (Figures 6b-d). These results suggest that the epididymal sperm number and quality were severely compromised on ES treatment.

### Exposure to ES in mice results in male infertility

**Figure 7:**
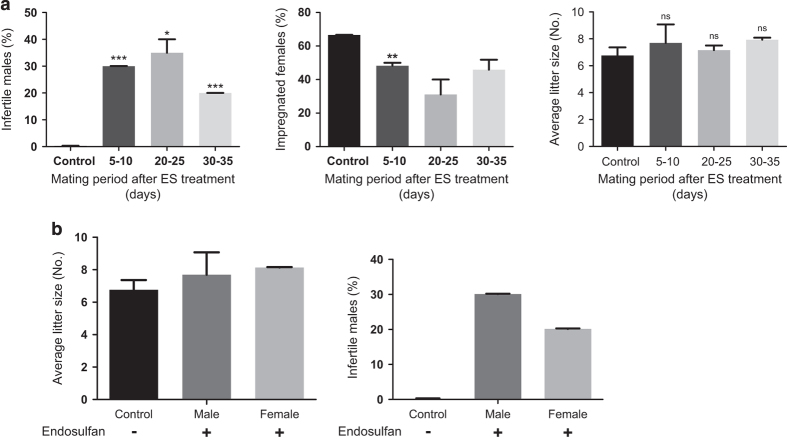
Evaluation of effect of Endosulfan on fertility in mice. (**a**) Bar graphs show average litter size and fertility in males and females at different mating intervals (5–10, 20–25 and 30–35 days) after ES treatment completion (3 mg/kg). (**b**) Comparison of difference in fertility levels when ES was given only to males (*n*=10) or to both males and females (*n*=10 each) (3 mg/kg). Mating window is 5–10 days after treatment completion. NS, not significant.

To test whether the effect on sperm count and motility affects the fertility in mice, mating experiments (1 : 3 ratio, male/female) were set up, post-ES treatment in males. Three mating windows (5–10, 20–25, 30–35 days post treatment of 3 mg/kg ES) covering one spermatogenesis cycle were selected. In all three mating windows, percentage of infertile males increased as opposed to no infertile males in control groups, indicating that the reduced sperm count and motility contributed towards reduced infertility in males (Figure 7a). Interestingly, the 20–25-day window showed maximum infertile males, supporting the reduced sperm count and motility at 25 days post treatment. Percentage of impregnated females followed a similar pattern, with a reduced number of pregnant females in 5–10- and 20–25-day-mating window (Figure 7a). However, there was no significant difference in average litter size among various groups belonging to different mating windows indicating that ES treatment in males might not result in implantation defects, which may lead to reduced litter size.

Since ES could affect male fertility, we wanted to test the synergistic effect of ES when both males and females are treated (3 mg/kg). Mating experiments were conducted in 1 : 1 ratio of males and females at 5–10 days post treatment. Numbers of infertile males were calculated from the number of non-impregnated females. Further increase in the number of infertile males (or non-impregnated females) was not observed as compared with the corresponding mating window, where only males were treated (Figure 7b). This suggests that there could be no synergistic effect on fertility when both males and females are exposed to ES. This also indicates that ES-induced infertility could be male specific (Figure 7b). Besides, average litter size showed no significant variation among different groups, further revealing that ES exposure may not contribute towards infertility in females. Thus, our results show that ES exerts effect on male reproductive system by depleting different testicular-cell populations, leading to reduced sperm count and motility, culminating in male infertility.

## Discussion

In the present study, using a variety of approaches we demonstrate that coupled with pathophysiological changes, ES induced tissue damage at an organismal level, exerting its effect maximally on testes causing cell death and depleting testicular-cell populations. ES-induced changes in testes were spermatogenesis dependent and led to the reduction in sperm count and motility resulting in male infertility.

### Effective concentration of ES used in mice is physiologically relevant

Selection of appropriate concentration of ES in the model system of the analysis, reflective of the concentration reported in humans in active area of exposure, is important to draw parallels between the effects in the model system and human beings. HPLC analysis of the serum from ES-treated mice revealed a sub-lethal dose of 20–25 *μ*g/l of ES within 24 h of treatment when 3 mg/kg of ES was administered to mice. This effective concentration is well within the range of concentration of ES or its derivatives found in human blood in the areas of active ES exposure (Supplementary Figure S1C) and below the lethal dose in humans (Supplementary Figure S1B). Therefore, the concentration which we have chosen for the study resembles that reported in humans and helps in the better extrapolation of physiological effects seen in mice.

### Specificity and selectivity of ES action

We have observed that ES induces weight fluctuations, no weight gains or high toxicity and lethality in males than females. We speculate this differential response of males and females to ES exposure could be associated with androgen receptor (AR) antagonist nature of ES. Several of the OCPs are found to be endocrine disruptors, acting as androgen or oestrogen antagonists. Previous studies have provided biochemical evidences to suggest ES as an AR antagonist, affecting gene expression in reporter-based assays.^24,25^ The natural abundance and distribution of AR in males^26^ may explain the higher selective response in males on ES exposure and higher toxicity of testes and lungs compared with other organs.

### ES exposure leads to infertility in males but not in females

Being an organ comprised of actively dividing cells and having high extent of pathophysiological damage induced by ES, we extensively analysed the fitness of spermatogenesis and its physiological consequences in testes from ES-exposed mice. Our results suggest that ES-induced damage in testes caused cell death in a spermatogenesis-dependent manner, affecting haploid, diploid and dividing mother cells over a complete spermatogenesis cycle, which was also reflected in the sperm quantity and quality and culminating in male infertility. Interestingly, unlike males, females did not show significant effect on exposure to ES, suggesting male-specific action of ES. Our results also provide an explanation for the observation of sperms having reduced competence in ES-exposed *Drosophila melanogaster*.^27^ Consistent to this, we did not detect any noticeable difference in body weight or physiology in females on exposure to ES, unlike males, suggesting that impact of ES in males is much more pronounced than females.

In conclusion, this is the first study that examines the mechanistic actions of a pesticide exerting pathophysiological changes, altering organ physiology, casing testicular damage and affecting testicular cells in a complete spermatogenesis cycle. A probable AR specificity further increases ES’ impact by its testes centric action and this damage further lead to qualitative and quantitative defects in spermatogenesis and results in male infertility.

## Materials and Methods

### Enzymes, chemicals and reagents

Chemicals and reagents were obtained from Sigma (St. Louis, MO, USA), Amresco (Solon, OH, USA) and Cisco Research Laboratories (Mumbai, India). ES (Pestinal) was obtained from Sigma.

### Animals

BALB/c mice or wistar rats of 6–8-weeks old were purchased from central animal facility, Indian Institute of Science, Bangalore, India and were maintained as per the guidelines of the animal ethical committee in accordance with Indian National Law on animal care and use. The animals were kept in polypropylene cages and provided standard pellet diet (Agro Corporation Pvt. Ltd., Bangalore, India) and purified water. The standard pellet diet is composed of 21% protein, 5% lipids, 4% crude fibre, 8% ash, 1% calcium, 0.6% phosphorus, 3.4% glucose, 2%vitamin and 55% nitrogen-free extract (carbohydrates). The animals were maintained under controlled conditions of temperature and humidity with a 12 h light/dark cycle.

### ES treatment in mice and rats

ES (3 mg/kg) was dissolved in 0.05% of methyl cellulose and mice were orally fed using a gastric gavage. The treatment consisted of four doses on every alternative day. All the experiments were performed with the consent from Indian Institute of Science animal ethics committee (Project No. CAF/Ethics/244/2011).

### Pharmacokinetics of ES

BALB/c mice were orally fed with ES (3 mg/kg, single dose). Animals were killed at 0.25, 0.5, 1, 2, 6, 12 and 24 h post administration and the sera were used for HPLC analysis.

### Flow cytometric analysis of mice lymphocytes

BALB/c mice, at the end of ES treatment (3 mg/kg) along with controls were killed, and bone marrow and thymus were collected (for CD19 and CD3 studies, 10 control and 13 treated mice were used). Blocking was done in 2% BSA and probed with anti-CD3-FITC, for thymus and CD19-APC for bone marrow. Cells were washed and analysed by flow cytometry (FACS Calibur, BD Biosciences, Franklin Lakes, NJ, USA). Using forward and side scatter, lymphocyte populations were gated and histogram was plotted using Flowing software (Turku, Finland) and the percentage of the gated cells was calculated and further used to plot vertical dot plots.

### Blood count analysis

BALB/c animals were killed along with controls after ES treatment (3 mg/kg, on 1, 11, 21 and 31 days post treatment) and blood was collected in a heparin-coated syringe by heart puncture and stored at 4 °C till use. RBC, WBC and platelet numbers were analysed within 2 h at Vijay Diagnostics, Bangalore, India.

### Liver and kidney function tests

Blood samples were collected from control and treated groups of BALB/c animals following treatment with ES (3 mg/kg, 1 and 21 days post treatment) using a non-heparinised syringe and allowed to clot at room temperature. After 1 h, sample was spun (6000 r.p.m., 10 min), plasma was collected and assayed for ALP, ALT, creatinine and urea using Autospan Kit (Span Diagnostic Ltd, Surat, India).

### Histopathological evaluation

Testes, liver, kidney, intestine, brain and lung tissues were dissected out from control and treated mice following treatment with ES (3 mg/kg) on 1st day after treatment completion. Tissues were processed for histological sectioning as described.^28–30^ Briefly, tissues were fixed in 4% paraformaldehyde, processed in xylene, embedded in paraffin wax, sectioned to 5–10 *μ*m in a rotary microtome (Leica Biosystems, Wetzlar, Germany) and stained with haematoxylin and eosin.^31^ Sections were then observed and photographed with light microscopy (Carl Zeiss, Zeiss, Oberkochen, Germany). Luxol blue staining was also performed on brain sections as described earlier.^32,33^

### TUNEL assay

TUNEL assay was performed (Calbiochem, San Diago, CA, USA) on testes and lung sections on 1st and 11th day after ES treatment (3 mg/kg). The tissue sections were de-paraffinized, rehydrated and permeabilized with proteinase K (2 mg/ml). Labelling reaction and detection was performed as per manufacturer’s directions. Images were obtained at ×20 and ×40 with Carl Zeiss Axioscope A1 light microscope (Germany).

### Testicular FACS analysis

Testicular FACS analysis was performed as described with modification.^34–36^ Briefly, ES-treated (3 mg/kg) and control animals (*n*=5 per group) were killed at respective time points (1, 11, 21 and 31 days post treatment) and the testes were washed in Hank’s Balanced Salt Solution and single cell suspension was prepared. Later, the cells were fixed at a dilution of ~5×10^6^ cells/ml using 70% chilled ethanol. Cells were washed, resuspended in PBS, added 1 *μ*g/ml of PI and analysed in BD FACSCanto (BD Biosciences), 10 min later. The data were analysed using Flowing software, and from the histogram, percentage of cells belonging to G_0_, 1n, G1, S and G2/M phases were calculated. Relative cell death was calculated by normalising G_0_ population of the treated group from a particular time point to the G_0_ population of respective control.

### Morphological analysis of epididymal sperm

Analysis of epididymal sperm was carried out as described previously.^37–39^ Following ES treatment (3 mg/kg, 11, 21 and 31 days post treatment), the animals were killed, epididymis was dissected out and placed in microfuge tube containing 1 ml TNE buffer (0.01 M Tris-HCl, 0.15 M NaCl, 1 mM EDTA) and mechanically disrupted to allow the sperms to swim out. After 2 min, the turbid solution containing sperms was taken without disturbing the sediment and diluted in TNE buffer to get a concentration of 1×10^7^ cells per ml. Equal amount of sperm cells and 1% Eosin Y solutions were mixed, smeared on to the slide and allowed to dry. Using DPX, a cover slip was mounted and observed under light microscope (Axioscope A1, Carl Zeiss) at a magnification of ×40. Morphological changes of head, hook and tail with respect to control sperms were noted for a number of 300 sperms per group.

### Sperm chromatin structure assay

SCSA was performed as described previously^40^ (*n*=5 per group). Dilution of epididymal sperm was made as described above. Sperm was used from mice belonging to 1, 11, 21 and 31 days post treatment completion of 3 mg/kg of ES, and for FACS analysis 0.6 ml of acid detergent (0.08 N HCl, 0.15 M NaCl, 0.1% Triton X-100) was added to 0.3 ml of diluted sperm in TNE buffer and incubated for 30 s. 1.2 ml of staining solution (0.1 M citric acid, 0.2 M Na_2_PO_4_, 1 mM EDTA, 0.15 M NaCl and 6 *μ*g/ml of acridine orange (AO)) was added to the mixture, incubated for 3 min and acquired in FACS. AO emits green fluorescence when bound to dsDNA, and red when bound to ssDNA or RNA. Since sperm head contains no RNA, a shift in red-high cells gives the measure of fragmented DNA in sperm. The red-high population was scored for the percentage of cells and plotted the cumulative percentage of red-high sperm in treated against that of control.

### Epididymal sperm ROS analysis

ROS level in epididymal sperm was assayed using DCFDA staining followed by FACS analysis.^41^ On the 1st day, 9 controls and 11 ES-treated (3 mg/kg) animals were killed, epididymis was dissected out and made dilution of 1X10^7^ sperms per ml in TNE buffer. DCFDA was added to a concentration of 10 *μ*M, incubated for 20 min at room temperature and analysed in BD FACSVerse (BD Biosciences).

### Sperm dispersion assay

Sperm dispersion assay was performed for the sperm sample which showed highest ROS levels (3 mg/kg, 1st day post-treatment completion) in epididymal sperm ROS analysis and for a control.^42^ Briefly, 30 *μ*l of sperm, diluted to 1X10^6^/ml was mixed with 70 *μ*l of low melting agarose and coated onto slides pre-coated with agarose. After solidification, the slides were subjected to 0.08 N HCl treatment for 17 min, lysis solution I (0.4 M Tris, 0.8 M DTT, 1% SDS and 50 mM EDTA) for 20 min, followed by lysis solution II (0.2 M Tris, 2 M NaCl, 1% SDS) for 15 min. The slides were then washed in TBE buffer, dehydrated in ethanol, stained using PI and visualised under Apotome fluorescence microscope (Carl Zeiss). Sperm halo was analysed as described.^43^ Quantification of the sperm halo area was done using ImageJ software (*n*=210 and 179 sperm heads, respectively, for control and treated group; NIH, Bethesda, MD, USA; http://imagej.nih.gov/ij/)).

### Clinical analysis of epididymal sperm

Clinical analysis of epididymal sperm was performed as described.^44^ Briefly, at respective time points (11, 26 and 36 days post 3 mg/kg ES treatment), treated animals were killed along with controls, the epididymis dissected out and homogenised in TNE buffer (1 ml), and allowed 3 min for the sperms to swim out (*n*=10 animals per group, at all time points). The sample was analysed immediately by the clinician at Infant laboratories, Bangalore, India, and percentage of actively motile, sluggish motile, non-motile sperms along with total sperm count were calculated.

### Mating experiments

Mating experiments in BALB/c mice were performed as described.^45^ For experiments involving treatment of males alone, males were treated and kept till the desired time window of mating (*n*=10, 3 mg/kg). Females of 6–8 weeks of age were used for experiments. At 5, 20 or 30 days post treatment, 1 : 3 (males/females) mating was set up in separate cages for 5 days. At the end of 5 days, males were separated from females and the females were observed for pregnancy in following days. A male was considered infertile if all three females in the cage failed to get impregnated. Average litter size was calculated, taking only the impregnated females into consideration. For experiments involving treatment of both males and females, animals were kept separately till 5 days, post treatment (*n*=10, 3 mg/kg). At 6th day post-treatment completion, 1 : 1 mating was set up in separate cages and animals were separated after 5 days. Here also, infertile male is defined by the number of non-impregnated females in the respective cage.

### Statistical analysis

All statistical analyses were performed using GraphPad Prism 5 software (La Jolla, CA, USA). For the analysis, if the data set followed normal distribution, a two-tailed, unpaired, student’s *t*-test was applied. If the data set did not follow normal distribution, a two-tailed Mann–Whitney test was applied for. Significance values were calculated as follows: *P<0.05, **P<0.01, ***P<0.001.
