# Supplementary material for: Exposure to Endosulfan can result in male infertility due to testicular atrophy and reduced sperm count
Source: Cell Death Discov. 2015 Nov 9;1:15020–. doi: 10.1038/cddiscovery.2015.20 (PMC4979443; doi:10.1038/cddiscovery.2015.20)
Supplement: Supplementary Information [file cddiscovery201520-s1.doc]

**Supplementary TEXT**

**Supplementary figure legends**

**Figure S1. Pathophysiological examination of mice treated with Endosulfan. A.** HPLC spectra profile of representative serum samples from 15 min, 1 h and 24 h after single ES oral feeding in mice (3 mg/kg). HPLC spectra of ES spiked in serum (20 µM) served as the control. **B.** Table describing the reports of concentrations of Endosufan in humans in clinical cases of ES poisoning. “n” represents the number of subjects in the given case. **C.** Table lists concentrations of Endosulfan present in blood or body fluids from humans lived in active area of exposure, reported in the literature). **D.** Vertical scatter plots indicating CD3^+^ population from thymus and CD19^+^ population from bone marrow following ES treatment completion (3 mg/kg). Numbers indicate the percentage of respective cell population in gated lymphocytes (Control n = 10, 11 and ES n = 13, 13; respectively for bone marrow and thymus).

**Figure S2. Histological evaluation of mice organs upon Endosulfan exposure. A.** Histopathology of intestine, brain and kidney of mice following ES treatment. Scale bar: 500 µm (10X). **B.** Luxol blue staining of ES treated brain sections. Scale bar: 500 µm (10X). In Fig. S1A and B, ‘Control’ indicates sections from no treatment animal and ‘ES’ is animals treated with Endosulfan (3 mg/kg).

**Figure S3. Analysis of spermatogenesis in mice upon Endosulfan treatment. A.** Cross section of seminiferous tubule of normal mouse stained with haematoxylin, showing different cell types. **B.** Testicular FACS analysis of control and ES treated animals at 1^st^, 11^th^, 21^st^ and 31^st^ day after ES treatment completion (3 mg/kg). n= 5 per group.

**References**

1. Boereboom FT, van Dijk A, van Zoonen P, Meulenbelt J. Nonaccidental endosulfan intoxication: a case report with toxicokinetic calculations and tissue concentrations. *J Toxicol Clin Toxicol* 1998; **36** (4)**:** 345-352.

2. Kucuker H, Sahin O, Yavuz Y, Yurumez Y. Fatal acute endosulfan toxicity: a case report. *Basic Clin Pharmacol Toxicol* 2009 Jan; **104** (1)**:** 49-51.

3. Eyer F, Felgenhauer N, Jetzinger E, Pfab R, Zilker TR. Acute endosulfan poisoning with cerebral edema and cardiac failure. *J Toxicol Clin Toxicol* 2004; **42** (6)**:** 927-932.

4. Blanco-Coronado JL, Repetto M, Ginestal RJ, Vicente JR, Yelamos F, Lardelli A. Acute intoxication by endosulfan. *J Toxicol Clin Toxicol* 1992; **30** (4)**:** 575-583.

5. Lino CM, da Silveira MI. Evaluation of organochlorine pesticides in serum from students in Coimbra, Portugal: 1997-2001. *Environ Res* 2006 Nov; **102** (3)**:** 339-351.

6. Arrebola FJ, Martinez Vidal JL, Fernandez-Gutierrez A. Analysis of endosulfan and its metabolites in human serum using gas chromatography-tandem mass spectrometry. *J Chromatogr Sci* 2001 May; **39** (5)**:** 177-182.

7. Martinez Vidal JL, Moreno Frias M, Garrido Frenich A, Olea-Serrano F, Olea N. Determination of endocrine-disrupting pesticides and polychlorinated biphenyls in human serum by GC-ECD and GC-MS-MS and evaluation of contributions to the uncertainty of the results. *Anal Bioanal Chem* 2002 Apr; **372** (7-8)**:** 766-775.

8. Cerrillo I, Granada A, Lopez-Espinosa MJ, Olmos B, Jimenez M, Cano A*, et al.* Endosulfan and its metabolites in fertile women, placenta, cord blood, and human milk. *Environ Res* 2005 Jun; **98** (2)**:** 233-239.

9. Freire C, Koifman RJ, Sarcinelli PN, Rosa AC, Clapauch R, Koifman S. Association between serum levels of organochlorine pesticides and sex hormones in adults living in a heavily contaminated area in Brazil. *Int J Hyg Environ Health* 2014 Mar; **217** (2-3)**:** 370-378.

10. Sosan MB, Akingbohungbe AE, Ojo IA, Durosinmi MA. Insecticide residues in the blood serum and domestic water source of cacao farmers in Southwestern Nigeria. *Chemosphere* 2008 Jun; **72** (5)**:** 781-784.
