# Supplementary material for: Exposure to Endosulfan can result in male infertility due to testicular atrophy and reduced sperm count
Source: Cell Death Discov. 2015 Nov 9;1:15020–. doi: 10.1038/cddiscovery.2015.20 (PMC4979443; doi:10.1038/cddiscovery.2015.20)
Supplement: Supplementary Figures [file cddiscovery201520-s2.ppt]

## Slide 1
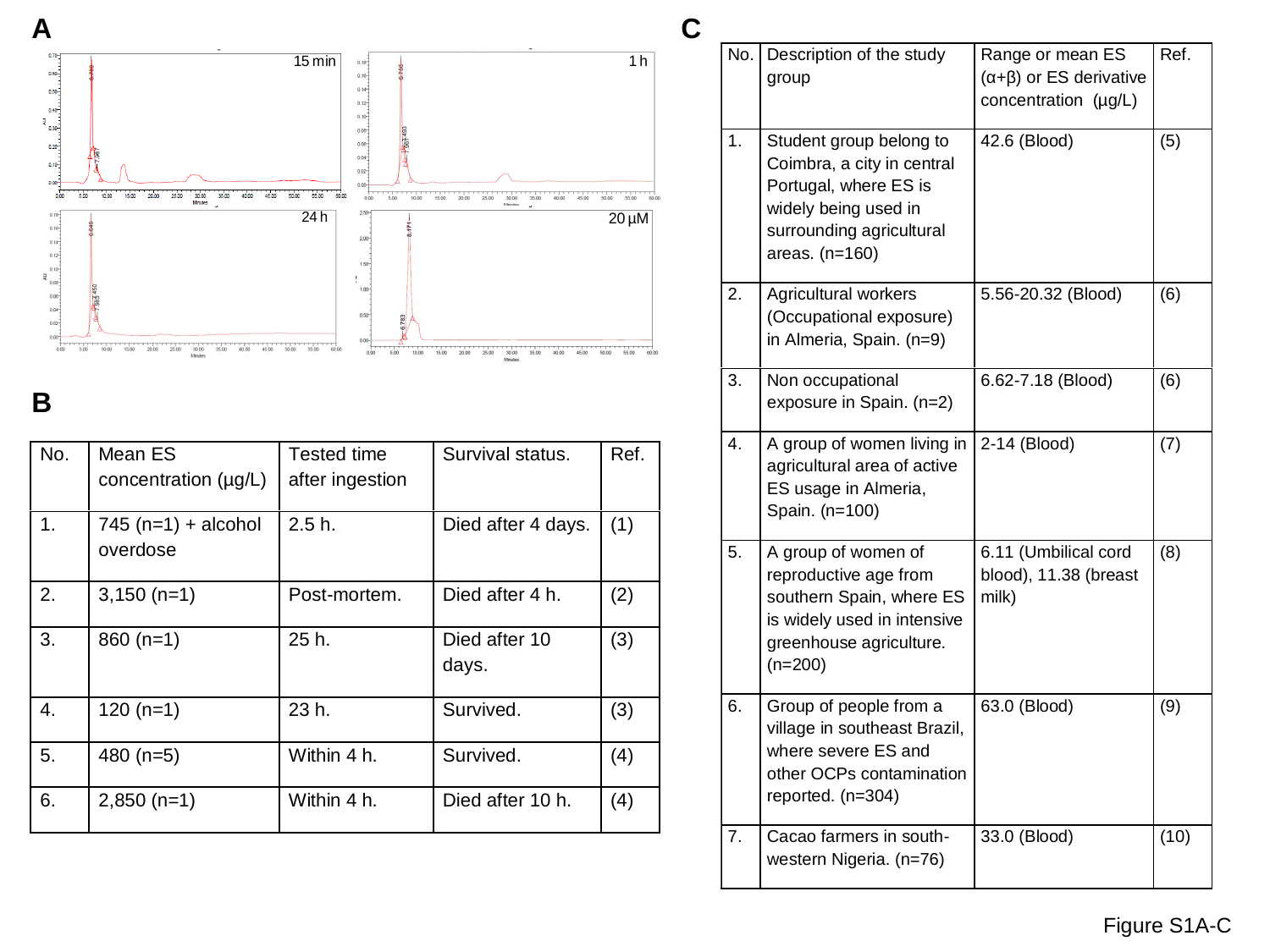

A
C
B
Figure S1A-C

## Slide 2
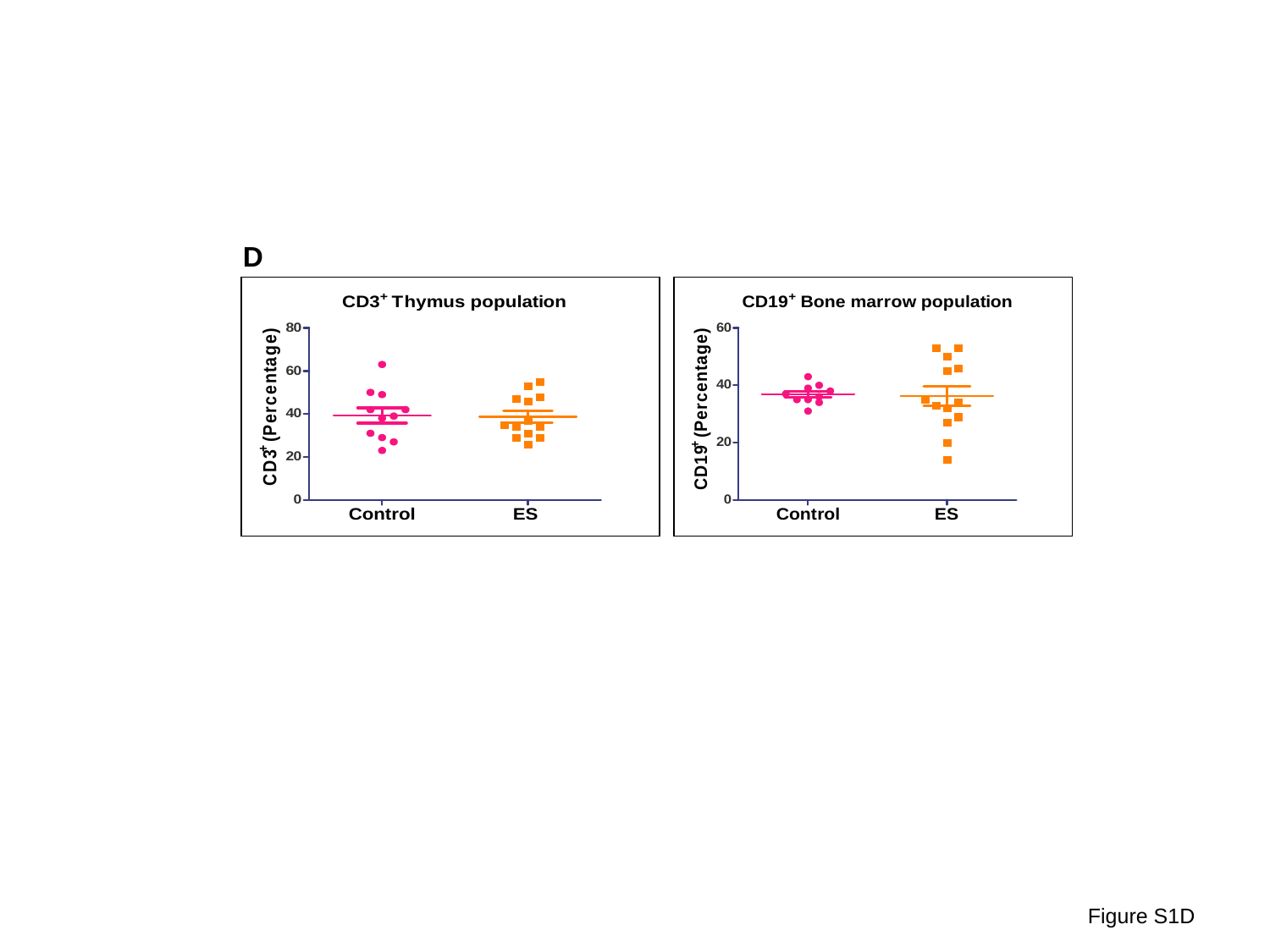

D
Figure S1D

## Slide 3
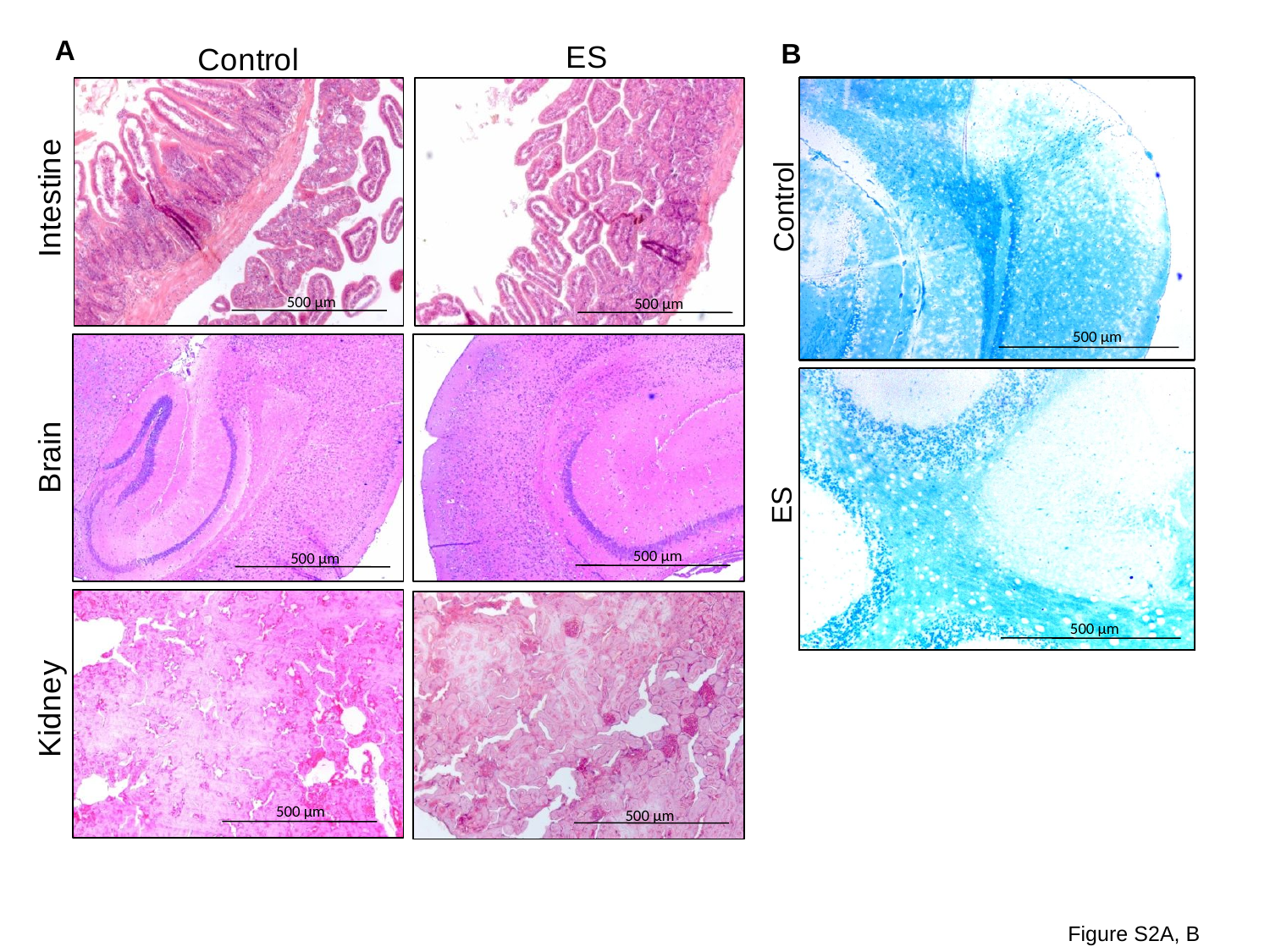

A
B
Control
ES
500 µm
500 µm
500 µm
500 µm
500 µm
500 µm
500 µm
500 µm
Figure S2A, B

## Slide 4
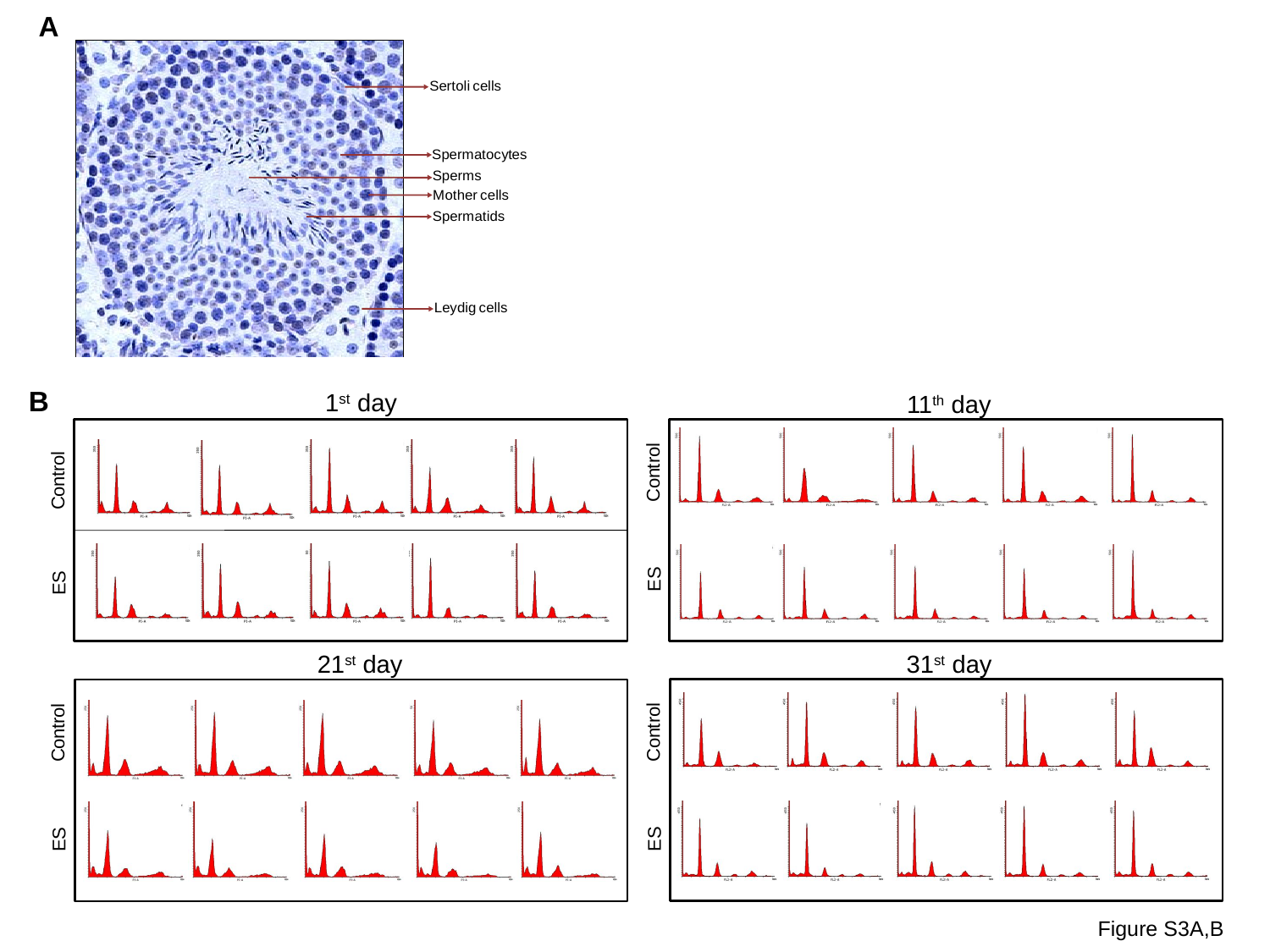

A
B
 1st day
11th day
31st day
21st day
Figure S3A,B
